# The effect of guideline-directed medicine on patients with new-onset heart failure following acute myocardial infarction

**DOI:** 10.3389/fcvm.2025.1639213

**Published:** 2025-09-19

**Authors:** Mengjie Lei, Jingyao Wang, Xiao Wang, Xue Sue, Cairong Li, Yanli Yang, Yachao Li, Zhigang Zhao, Zengming Xue

**Affiliations:** Department of Cardiology, Langfang People's Hospital Hebei, Langfang, China

**Keywords:** guideline-directed medical therapy, acute myocardial infarction, new-onset heart failure, prognosis, percutaneous coronary intervention

## Abstract

**Aims:**

To investigate the impact of guideline-directed medical therapy (GDMT) during hospitalization on the prognosis of patients with heart failure (HF) episode complicating post-acute myocardial infarction (AMI).

**Methods:**

From 01/05/2017 to 30/09/2022, 527 patients with HF episode complicating post-AMI at a single medical center who were retrospectively analyzed. Based on whether GDMT during hospitalization was used in patients undergoing percutaneous coronary intervention (PCI), the patients were divided into the GDMT group (*n* = 379) and the non-GDMT group (*n* = 148), with a follow-up period of 12 months after PCI. The primary endpoint was the composite endpoint of all-cause death and all-cause readmission.

**Results:**

The incidence of the primary endpoints (7.9% vs. 18.9%, *P* < 0.001), cardiac death and cardiac readmission composite events (5.5% vs. 15.5%, *P* = 0.002), all-cause readmission events (7.1% vs. 18.9%, *P* < 0.001), and cardiac readmission events (5.0% vs. 13.5%, *P* = 0.001) in the GDMT group were lower. Cox regression analysis revealed that the incidence of primary endpoints, cardiac death and cardiac readmission composite events, all-cause readmission events, and cardiac readmission events in patients treated with GDMT during hospitalization were 0.266 times (*HR* 0.266; *95% CI* 0.146–0.487; *P* < 0.001), 0.282 times (*HR* 0.282; *95% CI* 0.137–0.581; *P* = 0.001), 0.251 times (*HR* 0.251; *95% CI* 0.136–0.464; *P* < 0.001) and 0.262 times (*HR* 0.262; *95% CI* 0.125–0.551; *P* < 0.001), respectively, compared to patients treated without GDMT.

**Conclusion:**

For patients with HF episode complicating post-AMI who undergo PCI, the use of GDMT during hospitalization reduces the incidence of primary endpoints, cardiac death and cardiac readmission composite endpoints, and all-cause readmission and cardiac readmission.

## Introduction

Heart failure (HF), the final stage of cardiovascular disease, is a clinical syndrome caused by abnormal structure and/or function of the heart, leading to a decrease in cardiac output and/or an increase in intracardiac pressure during rest or loading, resulting in insufficient perfusion of tissues and organs (1). With the rapid aging of the population, the incidence of chronic diseases, such as coronary heart disease, hypertension, diabetes and obesity, is gradually increasing. The improvement of medical standards has prolonged the survival period of patients with cardiovascular disease, which has led to an increase in the prevalence of HF (2). The annual incidence rate of HF among European adults is approximately 5/1,000, and the prevalence of HF among adults is 1%–2% (3). It is generally believed that approximately 50% of hospitalized HF patients have heart failure with preserved ejection fraction (HFpEF)/heart failure with mildly reduced ejection fraction (HFmrEF) (4, 5). The proportion of ischemic causes is higher in heart failure (6). Ventricular remodeling after myocardial infarction is closely related to the occurrence and prognosis of HF, and the slowing or reversing of ventricular remodeling is an important factor in reducing the risk of death in heart failure patients after myocardial infarction. However, HF symptoms usually occur before ventricular remodeling. For patients with acute ST-segment elevation myocardial infarction (STEMI) and high-risk non-ST-elevation myocardial infarction (NSTEMI), early revascularization, rescue of dying myocardium, and reduction of the myocardial infarction area are important measures for preventing and treating ventricular remodeling (1). Guideline-directed medical therapy (GDMT) is an important means for acute myocardial infarction (AMI) patients to prevent ventricular remodeling and reduce readmission and mortality rates after revascularization. Currently, SGLT2i is recommended in heart failure-related guidelines for patients with different ejection fractions (I, A), and there is ample evidence to support the improvement of prognosis in HFrEF and HFmrEF patients with GDMT. For HFrEF patients, GDMTs are classified as Class I recommendations (I, A). For HFmrEF patients, except for SGLT2i, all other guideline-directed medicines are classified as Class IIb recommendations (IIb, C). For HFpEF patients, due to limited evidence, there is no clear recommendation for other guideline-directed medicine except for SGLT2i (1, 7), particularly in exploring its impact on the prognosis of HF episode complicating post-AMI. In addition, guidelines related to myocardial infarction indicate that regardless of left ventricular eject fraction (LVEF), conventional ACE inhibitors (IIa, A) should be considered for all ACS patients. Regardless of the HF symptoms, it is recommended that ACS patients with LVEF ≤ 40% use β receptor blockers (IIa, B) (8). Overall, for both heart failure guidelines and myocardial infarction guidelines, improving the prognosis of HF episode complicating post-AMI using GDMT still requires more clinical evidence, and some myocardial infarction patients in clinical practice are unable to use anti-ventricular remodeling drugs due to the impact of the infarct site on their heart rate and blood pressure. Therefore, this study explored the impact of GDMT on the prognosis of patients with HF episode complicating post-AMI who received percutaneous coronary intervention (PCI) to provide a reference for the timing of the clinical application of GDMT.

## Methods

### Study population

**Figure 1:**
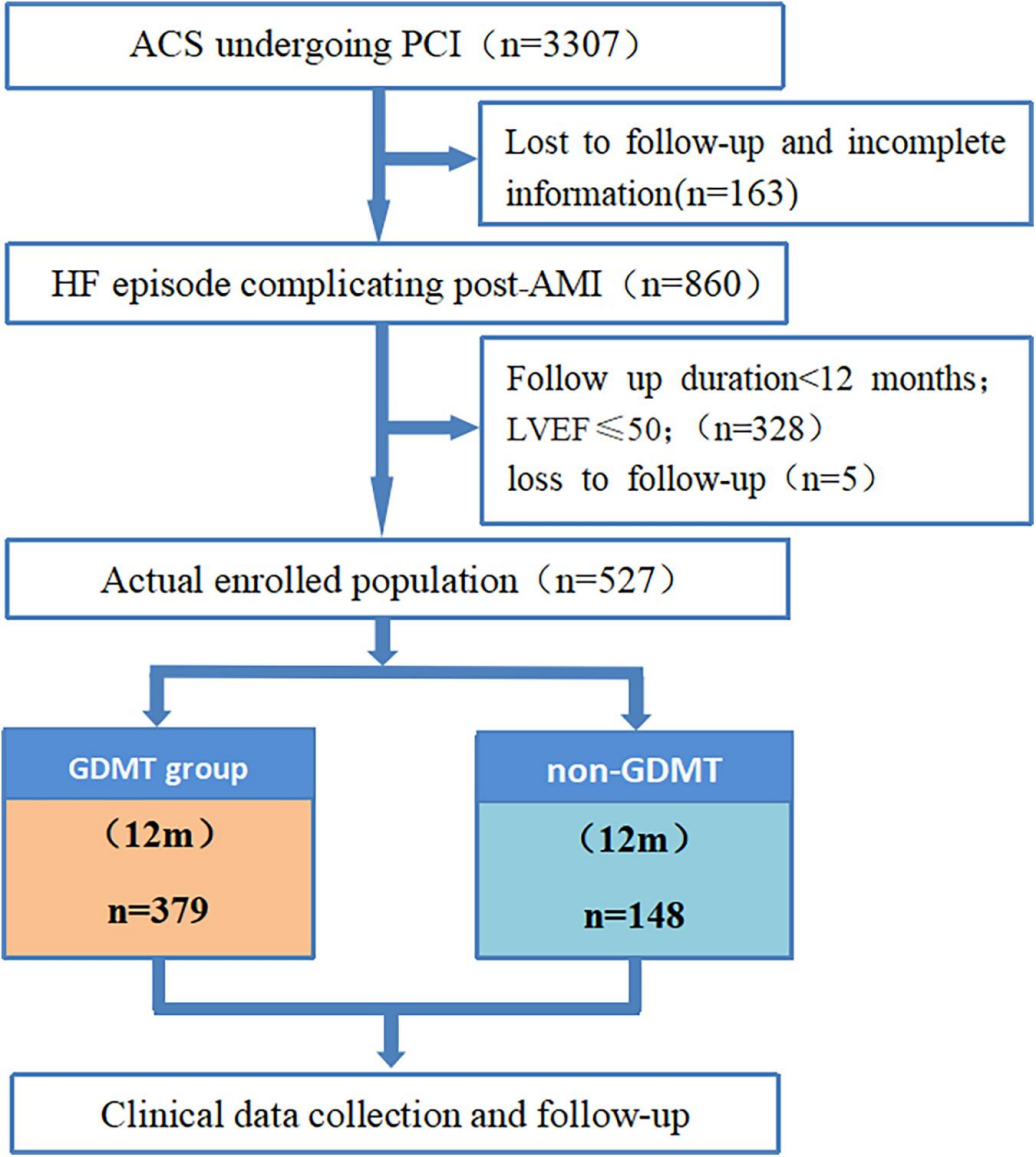
Flow chart of this study. ACS, acute coronary syndrome; PCI, percutaneous coronary intervention; AMI, acute myocardial infarction; HF, heart failure; HFmrEF, heart failure with mildly reduced ejection fraction; HFrEF, heart failure with reduced ejection fraction; GDMT, guideline-directed medical therapy.

This single-center retrospective cohort study included 527 patients with HF episode complicating who were hospitalized after AMI and had received PCI in the Department of Cardiology of Langfang People's Hospital from 01/05/2017 to 30/09/2022. Written informed consent from the patients/participants OR patients’/participants’ legal guardian/next of kin was not required to participate in this study in accordance with the national legislation and institutional requirements. According to whether GDMT was used during hospitalization, there were 379 patients in the GDMT group and 148 patients in the non-GDMT group. In addition, this study used cluster sampling to screen all PCI patients in our center, and Figure 1 shows the screening process for the target patients, which to some extent reduced selection bias. Diagnostic criteria: The patients included in this study were heart failure episode patients who underwent PCI after AMI and met the diagnostic criteria for both acute myocardial infarction (9). The inclusion criteria for patients were patients who had clear heart rate and blood pressure records during hospitalization; patients who had records for electrocardiography, NT-proBNP, echocardiography, and interventional treatment; patients who had detailed information on the use of GDMT (including cases where it was not applied due to contraindications); patients aged ≥18 years; and patients who were followed up for ≥12 months. The exclusion criteria for patients were as follows: previously diagnosed with heart failure or other causes of heart failure, such as heart failure caused by cardiomyopathy; LVEF ≤ 50%; elevated NT-proBNP caused by extracardiac factors such as hyperthyroidism, sepsis, stroke, or pulmonary disease; combined severe cognitive impairment; currently participating in other similar studies; expected lifespan less than 1 year; and severe liver and kidney dysfunction (Child‒Pugh grade 2–3 or eGFR < 30 ml/min/1.73 m^2^).

### Baseline data collection

A self-designed case report form containing baseline data and prognostic follow-up results for the patient was used. The baseline data of patients were collected by consulting electronic medical records. Patient grouping and prognostic follow-up data were obtained, and the patients completed the case report form via telephone and outpatient follow-up. After the completion of the case report, the relevant data were entered into SPSS 26.0 data software, and data entry was carried out by two people to ensure accuracy. The baseline data in this study were determined after extensive literature review and discussion, including general patient information and diagnosis and treatment strategies during hospitalization, such as age, sex, body mass index, myocardial infarction classification, primary PCI, LVEF, location of myocardial infarction, heart rate and blood pressure at discharge, previous history, laboratory tests, etc. The primary endpoint was the composite endpoint of all-cause mortality and all-cause readmission during follow-up. The secondary endpoints were the composite endpoints of cardiac death and cardiac readmission, all-cause mortality, cardiac death, all-cause readmission, and cardiac readmission. The follow-up period was 12 months after PCI.

### Definitions

In this study, patients in the GDMT group who underwent PCI during hospitalization were treated with GDMT [at least one renin angiotensin aldosterone system inhibitor (RAAS inhibitor), β receptor blocker, mineralocorticoid receptive antagonist (MRAs), or sodium-dependent glucose transporter 2 inhibitor (SGLT2i)] for 12 months. Other treatment options were the same as those in the no-GDMT group. The patients in the no-GDMT group did not receive GDMT (any of the RAAS inhibitors, B receptor blockers, MRAs, or SGLT2i) after PCI during hospitalization.

All-cause mortality: the ratio of the total number of deaths occurring for various reasons within one year after PCI to the total number of people included in the study.

The causes of cardiac death were as follows: (1) death caused by cardiac shock or heart failure; (2) death caused by complications after acute myocardial infarction, such as ventricular septal perforation, cardiac tamponade, or cardiac rupture; (3) death caused by malignant arrhythmia; and (4) death related to PCI.

All-cause readmission: The ratio of the total number of patients admitted for various reasons within one year after PCI to the total number of patients included in the study.

Cardiac readmission: The ratio of the number of cardiac readmissions in patients one year after PCI to the total number of patients included in the study.

### Follow-up

The study population was drawn from our prospective PCI database, in which data were collected prospectively and tracked by trained full-time data officers. This approach helped reduce recall bias regarding the outcome measures to some extent. The outpatient follow-up times were 2 weeks, 3 months, 6 months, and 1 year after PCI. Patients who had not undergone outpatient follow-up were followed up by phone. The main content of the follow-up was the patient's medication adherence and the occurrence of endpoint events. The types and conventional dosages of medications used by patients in this study are presented in Table S1.

### Statistical analysis

Statistical analysis was conducted using SPSS 26.0 software. Normally distributed measurement data are presented as the mean ± standard deviation, and intergroup comparisons were analyzed using independent sample *t*-tests. Nonnormally distributed measurement data are presented as the median and interquartile range, and intergroup comparisons were analyzed using rank sum tests. Count data are expressed as frequencies and percentages, and the chi-square test was used to analyze differences between groups. Cox multivariate regression analysis was used to adjust the baseline data. The Kaplan–Meier method was used to analyze the survival rates of patients in the two groups. Subgroup analysis was implemented using Stata 17.0 software. Differences were considered statistically significant at *P* < 0.05.

## Results

### Baseline characteristics

There were no significant differences between the two groups in terms of age, sex, STEMI status, primary PCI ratio, acute myocardial infarction site, door-to-balloon time, first medical contact-to-balloon time, heart rate at discharge, systolic blood pressure at discharge, hypertension history, type 2 diabetes history, cerebrovascular disease history, old myocardial infarction, smoking history, family history of coronary heart disease, history of peripheral artery disease, history of complete revascularization or history of chronic obstructive pulmonary disease according to the baseline data (*P* > 0.05). The body mass index (BMI) of patients in the GDMT group was significantly greater than that in the non-GDMT group (*P* < 0.001). The left ventricular ejection fraction (LVEF) of patients in the GDMT group was significantly lower than that in the non-GDMT group (*P* = 0.003). Compared with patients in the GDMT group, patients in the non-GDMT group had a significantly greater incidence of atrial fibrillation (*P* = 0.034). The application rates of RAAS inhibitors, β receptor blockers, MRAs, and SGLT2i in all patients were 33.0%, 55.2%, 29.8%, and 2.3%, respectively. The application rates of RAAS inhibitors, β receptor blockers, MRAs, and SGLT2is in patients treated with GDMT were 45.9%, 76.8%, 41.4%, and 3.2%, respectively. There was a significant difference in the application rates of RAAS inhibitors, β receptor blockers, MRAs, and SGLT2is between patients in the GDMT group and those in the non-GDMT group (*P* < 0.001, *P* < 0.001, *P* < 0.001, *P* = 0.005) (Table 1).

**Table 1:** Baseline characteristics.

Characteristic	GDMT group (*n* = 379)	Non-GDMT group (*n* = 148)	*F*/*χ²* value	*P* value
Age (years; m ± SD)	60.14 ± 11.58	61.53 ± 10.61	1.267	0.206
Female (*n*, %)	113 (29.8%)	47 (31.8%)	0.190	0.663
BMI (kg/m^2^; m ± SD)	26.22 ± 3.51	24.92 ± 3.14	3.934	<0.001
STEMI (*n*, %)	314 (82.8%)	124 (83.8%)	0.066	0.797
Primary PCI (*n*, %)	230 (60.7%)	92 (62.2%)	0.098	0.755
Complete RV (*n*, %)	181 (47.8%)	68 (45.9%)	0.140	0.708
LVEF	57.59 ± 3.21	58.54 ± 3.29	3.035	0.003
Anterior wall series myocardial infarction (*n*, %)	184 (48.5%)	75 (50.7%)	0.193	0.661
Heart rate at discharge (bpm, m ± SD)	72.04 ± 10.26	71.61 ± 8.35	0.493	0.622
Heart rate at discharge ≤65 bpm (*n*, %)	86 (22.7%)	23 (15.5%)	3.318	0.661
Systolic blood pressure at discharge (mmHg, m ± SD)	122.67 ± 16.87	123.71 ± 14.65	10.660	0.510
Systolic blood pressure at discharge ≤130 mmHg (*n*, %)	282 (74.4%)	103 (69.6%)	0.152	0.263
D to B time (minute, m ± SD)	96.73 ± 22.42	96.77 ± 22.46	0.024	0.981
FMC to B time (minute, m ± SD)	117.25 ± 40.06	114.52 ± 35.29	0.724	0.469
Medical history (*n*, %)				
Hypertension	256 (67.5%)	99 (66.9%)	0.021	0.886
Type 2 diabetes	81 (21.4%)	27 (18.2%)	0.639	0.424
Cerebrovascular disease	48 (12.7%)	15 (10.1%)	0.647	0.421
OMI	22 (5.8%)	6 (4.1%)	0.648	0.421
Atrial fibrillation	4 (1.1%)	6 (4.1%)	4.515	0.034
Current smoker	185 (48.8%)	81 (54.7%)	1.491	0.222
CAD family history	12 (3.2%)	4 (2.7%)	0.079	0.778
Peripheral artery disease	1 (0.3%)	1 (0.7%)	–	0.483
COPD	0 (0.0%)	2 (1.4%)	–	0.078
RAAS inhibitors (*n*, %)	174 (45.9%)	0 (0.0%)	101.440	<0.001
β receptor blocker (*n*, %)	291 (76.8%)	0 (0.0%)	253.755	<0.001
MRAs (*n*, %)	157 (41.4%)	0 (0.0%)	87.323	<0.001
SGLT2i (*n*, %)	12(3.2%)	0(0.0%)	8.021	0.005

GDMT, guideline-directed medical therapy; BMI, body mass index; STEMI, ST segment elevation myocardial infarction; PCI, percutaneous coronary intervention; RV, revascularization; LVEF, left ventricular ejection fraction; D to B time, door to balloon time; FMC to B time, first medical contact to balloon time; OMI, old myocardial infarction; CAD, coronary artery disease; COPD, chronic obstructive pulmonary disease; RAAS inhibitors, renin angiotensin aldosterone system inhibitors; MRAs, mineralcorticoid recept antagonist; SGLT2i, sodium-dependent glucose transporters 2 inhibitor.

### Laboratory tests

There was no significant difference between the two groups in terms of hemoglobin (HGB), white blood cell count (WBC), fibrinogen, cardiac troponin I (cTnI), N-terminal B type natriuretic peptide (NT-proBNP), creatine kinase isoenzymes (CK-MB), renal function, glycosylated hemoglobin, blood lipids, etc. (*P* > 0.05). The platelet counts of patients in the GDMT group were significantly greater than those in the non-GDMT group (*P* = 0.026). The fasting blood glucose levels of patients in the GDMT group were significantly lower than those in the non-GDMT group (*P* = 0.050) (Table 2).

**Table 2:** Laboratory tests.

Characteristic	GDMT group (*n* = 379)	Non-GDMT group (*n* = 148)	*F*/*χ²* value	*P* value
HGB (g/L)	138.27 ± 16.42	135.44 ± 23.21	1.355	0.177
WBC (×10^9^/L)	8.50 ± 2.57	8.57 ± 2.85	0.258	0.797
PLT (×10^9^/L)	242.25 ± 54.46	230.13 ± 59.78	2.232	0.026
FIB (g/L)	3.46 ± 0.92	3.37 ± 0.88	0.995	0.320
cTnI (µg/L)	4.70 (0.31, 19.76)	6.30 (0.91, 15.00)	1.250	0.211
NT-pro BNP (ng/L)	918.00 (574.00, 1,711.00)	1,023.50 (530.75, 1,655.50)	0.516	0.606
CK-MB (U/L)	29.00 (11.00, 92.62)	20.00 (11.00, 71.95)	1.381	0.167
Cr (µmol/L)	69.54 ± 19.17	66.18 ± 18.17	1.830	0.068
eGFR	94.07 ± 18.77	95.28 ± 17.05	0.681	0.496
UA (µmol/L)	333.37 ± 93.07	331.27 ± 91.17	0.235	0.815
FBG (mmol/L)	6.54 ± 2.04	6.94 ± 2.27	1.962	0.050
HbA1C (%)	6.91 ± 2.07	6.78 ± 1.11	0.234	0.815
TC (mmol/L)	4.95 ± 1.34	5.16 ± 1.77	1.221	0.224
TG (mmol/L)	1.93 ± 1.19	1.85 ± 1.26	0.684	0.494
LDL-C (mmol/L)	2.93 ± 0.88	3.08 ± 1.03	1.542	0.124
HDL-C (mmol/L)	0.90 ± 0.31	0.83 ± 0.54	1.638	0.102
Lp (a) (mg/L)	332.82 (120.20, 332.82)	332.82 (239.23, 332.82)	1.518	0.129

GDMT, guideline-directed medical therapy; HGB, haemoglobin; WBC, white blood cell count; PLT, platelet; FIB, fibrinogen; cTnI, cardiac troponin; NT-pro BNP, N terminal B type natriuretic peptide; CK-MB, creatine kinase isoenzymes; Cr, Serum creatinine; eGFR, estimated glomerular filtration rate; UA, uric acid; FBG, fasting blood glucose; HbA1C, glycosylated hemoglobin; TC, total cholesterol; TG, triglyceride; LDL-C, low density lipoprotein-cholesterol; HDL-C, high density lipoprotein-cholesterol; Lp (a), Lipoprotein (a).

### Coronary angiography during hospitalization

There was no significant difference between the two groups in terms of ostial lesion, diffused lesion, chronic total occlusion, complete revascularization (*P* > 0.05). The proximal segment of left anterior descending of patients in the GDMT group were significantly higher than that in the non-GDMT group (*P* = 0.026) (Table 3).

**Table 3:** Coronary angiography.

Characteristic	GDMT group (*n* = 379)	Non-GDMT group (*n* = 148)	*F*/*χ²* value	*P* value
LADp (*n*, %)	65 (17.2%)	14 (9.5%)	4.940	0.026
Ostial lesion (*n*, %)	113 (29.8%)	44 (29.7%)	0.000	0.985
Diffused lesion (*n*, %)	117 (46.7%)	68 (45.9%)	0.024	0.876
CTO (*n*, %)	14 (3.7%)	2 (1.4%)	1.268	0.655
Complete RV (*n*, %)	181 (47.8%)	68 (45.9%)	0.140	0.708

GDMT, guideline-directed medical therapy; GDMT group, the patients in the GDMT group received GDMT after Percutaneous coronary intervention during hospitalization (at least one RAAS inhibitor, β receptor blocker, MRA, or SGLT2i) for 12 months; non-GDMT group, the patients in the non-GDMT group did not receive GDMT (any RAAS inhibitor, β receptor blocker, MRA, or SGLT2i) after PCI during hospitalization; LADp proximal segment of left anterior descending; CTO, chronic total occlusion; RV, revascularization.

### Endpoints during follow-up

The analysis results showed that compared with patients in the GDMT group, patients in the non-GDMT group had a significantly greater incidence of all-cause death and all-cause readmission composite endpoint events, cardiac death and cardiac readmission composite endpoint events, and all-cause readmission and cardiac readmission events during follow-up (*P* < 0.001, *P* = 0.002, *P* < 0.001, *P* = 0.001). During the follow-up period, there was no statistically significant difference in the incidence of all-cause mortality, cardiac death, heart failure readmission, stroke or revascularization between the two groups of patients (*P* > 0.05 for both) (Table 4).

**Table 4:** Endpoints during follow-up.

Characteristic	GDMT group (*n* = 379)	Non-GDMT group (*n* = 148)	*χ²* value	*P* value
All-cause mortality and all-cause readmission composite endpoint (*n*, %)	30/379 (7.9%)	28/148 (18.9%)	13.157	<0.001
Cardiac death and cardiac readmission composite endpoint (*n*, %)	21/379 (5.5%)	20/148 (15.5%)	9.430	0.002
All-cause mortality (*n*, %)	4/379 (1.1%)	0/148 (0.0%)	0.485	0.486
Cardiac death (*n*, %)	1/379 (0.3%)	0/148 (0.0%)	–	1.000
All-cause readmission (*n*, %)	27/379 (7.1%)	28/148 (18.9%)	15.841	<0.001
Cardiac readmission (*n*, %)	19/379 (5.0%)	20/148 (13.5%)	11.223	0.001
Readmission for heart failure (*n*, %)	4/379 (1.1%)	1/148 (0.7%)	0.175	0.676
Stroke (*n*, %)	1/379 (0.3%)	3/148 (2.0%)	2.364	0.124
Revascularization (*n*, %)	11/379 (2.9%)	9/148 (6.1%)	2.946	0.086

GDMT, guideline-directed medical therapy; HF, heart failure.

### Results of cox multivariate regression analysis

After incorporating variables with significant differences at baseline (BMI, LVEF, history of atrial fibrillation, platelet count, fasting blood glucose level, and proximal segment of left anterior descending) and related influencing factors clinically believed to interfere with the study results as independent variables into the regression equation, the incidence of all-cause mortality and all-cause readmission composite events, incidence of cardiac mortality and cardiac readmission composite events, incidence of all-cause readmission events and incidence of cardiac readmission events in patients in the GDMT group were 0.266 times (*HR* 0.266; *95% CI* 0.146–0.487; *P* < 0.001), 0.282 times (*HR* 0.282; *95% CI* 0.137–0.581; *P* = 0.001), 0.251 times (*HR* 0.251; *95% CI* 0.136–0.464; *P* < 0.001), and 0.262 times (*HR* 0.262; *95% CI* 0.125–0.551; *P* < 0.001), respectively, greater than those in patients in the non-GDMT group. The incidence of cardiac mortality and cardiac readmission composite events and incidence of cardiac readmission events in patients with a history of atrial fibrillation were 4.644 times higher (*HR* 4.644; *95% CI* 1.398–15.426; *P* = 0.012) and 4.787 times higher (*HR* 4.787; *95% CI* 1.438–15.934; *P* = 0.011) than those in patients without a history of atrial fibrillation. The Cox regression analysis results revealed that BMI, LVEF, platelet count, and fasting blood glucose were not significantly different between the two groups (Table 5).

**Table 5:** Cox regression.

Outcome	Variable	Quotient		*Wald*	*HR*	95% CI	*P* value
		*B*	*SE*				
All-cause mortality and all-cause readmission composite events	GDMT	−1.323	0.308	18.498	0.266	0.146–0.487	<0.001
Cardiac death and cardiac readmission composite events	GDMT	−1.266	0.368	11.806	0.282	0.137–0.581	0.001
	Atrial fibrillation	1.536	0.613	6.284	4.644	1.398–15.426	0.012
All-cause readmission	GDMT	−1.382	0.313	19.445	0.251	0.136–0.464	<0.001
Cardiac readmission	GDMT	−1.339	0.379	12.505	0.262	0.125–0.551	<0.001
	Atrial fibrillation	1.566	0.614	6.513	4.787	1.438–15.934	0.011

GDMT, guideline-directed medical therapy.

### Kaplan–Meier survival analysis curves

**Figure 2:**
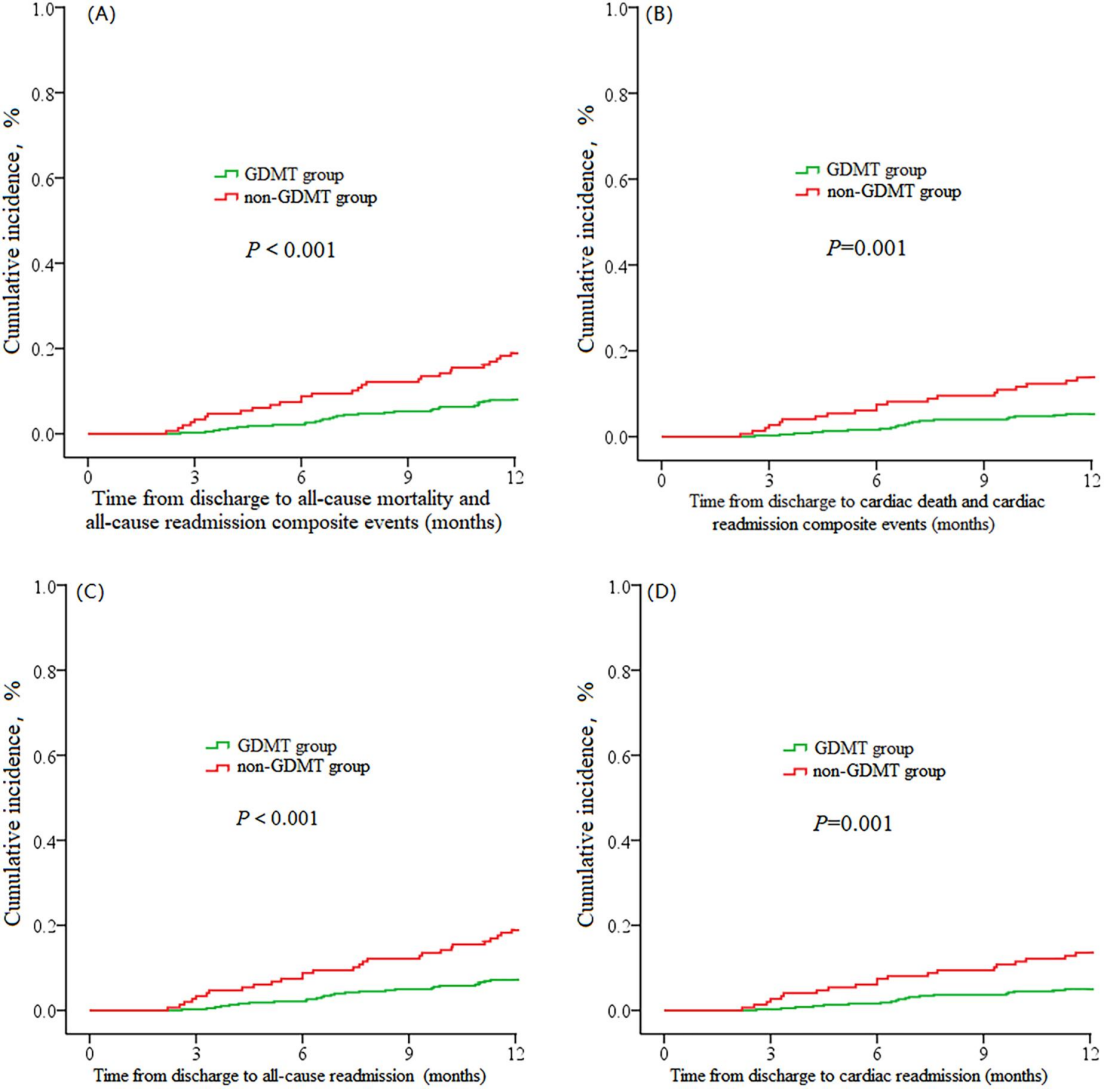
**(A)** The cumulative incidence of all-cause mortality and all-cause readmission composite events. **(B)** The cumulative incidence of cardiac death and cardiac readmission composite events. **(C)** The cumulative incidence of all-cause readmission events. **(D)** The cumulative incidence of cardiac readmission events.

The Kaplan‒Meier survival curve showed that the incidence of all-cause death and all-cause readmission composite endpoint events, cardiac death and cardiac readmission composite endpoint events, and all-cause readmission and cardiac readmission events within 1 year in the GDMT group were significantly lower than those in the non-GDMT group (*P* < 0.001, *P* = 0.002, *P* < 0.001, *P* = 0.001) (Figure 2).

### Subgroup analysis of the two groups of patients

**Figure 3:**
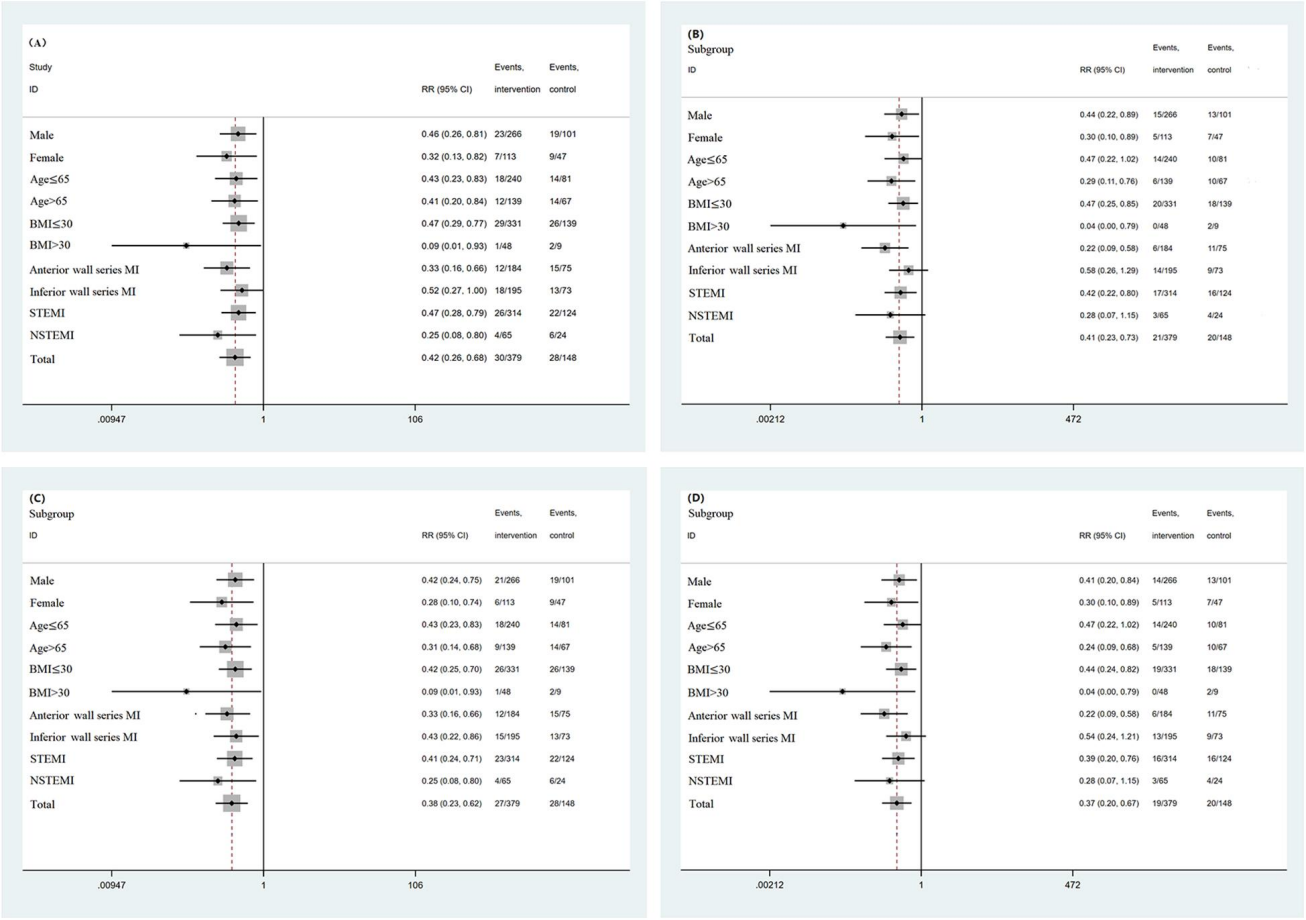
**(A)** Subgroup analysis based on baseline data (all-cause mortality and all-cause readmission composite events). **(B)** Subgroup analysis based on baseline data (cardiac death and cardiac readmission composite events). **(C)** Subgroup analysis based on baseline data (all-cause readmission events). **(D)** Subgroup analysis based on baseline data (cardiac readmission events).

**Figure 4:**
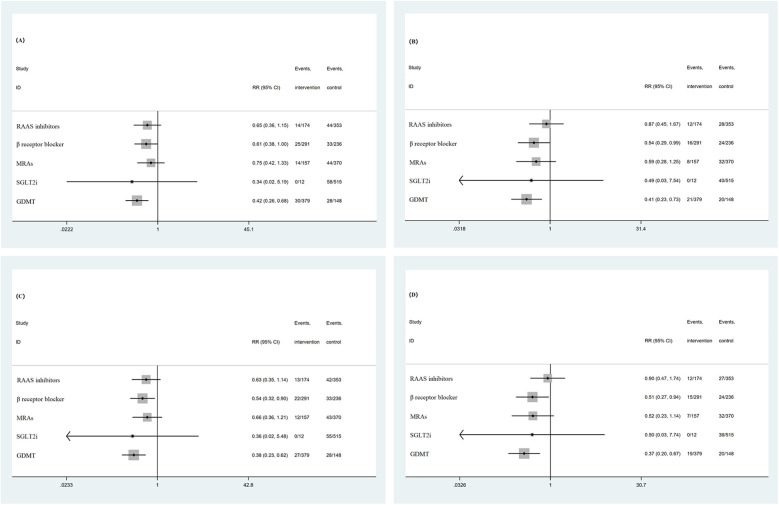
**(A)** Subgroup analysis based on medication regimen (all-cause mortality and all-cause readmission composite events). **(B)** Subgroup analysis based on medication regimen (cardiac death and cardiac readmission composite events). **(C)** Subgroup analysis based on medication regimen (all-cause readmission events). **(D)** Subgroup analysis based on medication regimen (cardiac readmission events).

Subgroup analysis was conducted based on sex, age, BMI, location of myocardial infarction, and myocardial infarction subtype. For all-cause mortality, all-cause readmission composite events and all-cause readmission events, the results of each subgroup analysis were consistent with the overall results. For the composite endpoint events of cardiac death and cardiac readmission, as well as cardiac readmission, except for the lower wall series myocardial infarction subgroup and NSTEMI subgroup, the overall results of the other subgroups were consistent. For the lower wall series myocardial infarction subgroup and NSTEMI subgroup, the incidence of events in patients treated with GDMT was significantly lower than that in patients not treated with GDMT (*P* < 0.05). The incidence of all-cause mortality and all-cause readmission composite events and the incidence of all-cause readmission events in patients treated with GDMT were significantly lower than those in patients not treated with GDMT (*P* < 0.05). Subgroup analysis was performed according to whether the patient was receiving RAAS inhibitors, β receptor blockers, MRAs, or SGLT2is. The results showed that the incidence of all-cause readmission and cardiogenic readmission in patients treated with β receptor blockers was significantly lower than that in patients treated without β receptor blockers (*P* < 0.05). However, there was no significant difference in the incidence of all-cause death and all-cause readmission composite events, cardiac death and cardiac readmission composite events, all-cause readmission, or cardiac readmission between the patients who were treated with RAAS inhibitors, MRAs, or SGLT2is and those who were not treated (*P* > 0.05) (Figures 3, 4).

## Discussion

This was a real-world single-center cohort study that retrospectively analyzed 527 patients with HF episode complicating post-AMI who underwent PCI. The main findings were as follows: (1) In terms of the application of GDMT in this study, the application rates of RAAS inhibitors, β receptor blockers, MRAs, and SGLT2i were lower in patients with HFpEF after AMI. (2) For patients with HF episode complicating post-AMI undergoing PCI, compared to patients in the non-GDMT group, patients who initiated GDMT in the hospital had lower rates of all-cause mortality and all-cause readmission composite events, cardiac death and cardiac readmission composite events, and all-cause readmission events and cardiac readmission events; (3) GDMT was the factor influencing all-cause mortality and all-cause readmission composite events and all-cause readmission events in HF episode complicating post-AMI. GDMT and a history of atrial fibrillation were factors influencing cardiac death and cardiac readmission composite events and cardiac readmission in HF episode patients after AMI; (4) The subgroup analysis (sex, age, BMI, location of myocardial infarction, and myocardial infarction subtype) showed that the results for the different subgroups were basically consistent with the overall results of this study. (5) The exploratory analysis revealed that all individual GDMT agents exhibited a consistent trend of risk reduction, and in-hospital initiation of ≥1 GDMT agent conferred significant benefits to patients compared with the complete absence of GDMT initiation.

Although there is still a lack of clear evidence of treatment methods to improve ventricular remodeling in patients with HF episode complicating post-AMI, many doctors in clinical practice are gradually accepting the use of RAAS inhibitors, β receptor blockers or MRAs to treat most HF episode patients with concomitant hypertension and/or CHD. At baseline, in the PARAGON-HF study, over 86% of patients used ACEIs/ARBs, 80% of patients used β receptor blockers, and over 24% of patients used MRAs (10). However, the application rates of RAAS inhibitors, β receptor blockers, MRAs, and SGLT2i in this study were relatively low, at 33.0%, 55.2%, 29.8%, and 2.3%, respectively. On the one hand, for both heart failure guidelines and myocardial infarction guidelines, improving the prognosis of HF episode complicating post-AMI using GDMT still requires more clinical evidence. Therefore, clinicians may rely more on their own treatment habits to decide whether to apply GDMT to patients. the awareness of GDMT in clinical practice may still need to be improved, and on the other hand, GDMT depends on the blood pressure, heart rate, and estimated glomerular filtration rate (eGFR) of patients (11). When the patient's hemodynamics are unstable, complicates GDMT initiation compared to HF from other etiologies, requiring physicians to balance potential benefits against stability concerns. In addition, due to the short marketing time of SGLT2is in China, although SGLT2is have significant benefits in patients with various types of heart failure compared to other guideline-guided medications, the application rate of SGLT2is in this study was only 2.3%. Considering that the medication regimen for patients at discharge may be influenced by their blood pressure and heart rate at discharge, the baseline blood pressure and heart rate of two groups of patients at discharge were analyzed in this study, and the results showed no statistically significant differences.

In addition, for patients with AMI, there may be myocardial stunning, also known as postischemic myocardial dysfunction, which refers to the temporary myocardial ischemia phenomenon that has not yet caused myocardial necrosis, but mechanical dysfunction takes several hours, days, or weeks to fully recover after reperfusion to restore normal blood flow (12). When myocardial stunning occurs, the temporary decrease in blood pressure caused by the inability of some areas of the myocardium to immediately resume normal contraction is also one of the reasons why patients cannot initiate GDMT early in the hospital. This finding also suggests that compared to other HF patients, HF patients with AMI are more likely to experience dynamic changes in LVEF after discharge (13, 14). For patients with different ejection fractions, the guidelines have different recommendations for GDMT. Therefore, dynamic evaluation and follow-up of heart failure patients after AMI are particularly important, especially using echocardiography to objectively measure multiple indicators reflecting systolic and diastolic function. It should be noted that heart failure with improved ejection fraction or heart failure with recovered ejection fraction only represents a certain degree of restoration of cardiac function or structure and is not a cure or complete normalization. The use of GDMT should continue to improve the disease prognosis.

In terms of SGLT2i alone, studies by DELEVER and EMPEROR have shown that the prognosis of patients with various types of heart failure can improve (15, 16). The 2022 AHA heart failure guidelines indicate that SGLT2i can improve the prognosis of HFpEF and HFmrEF patients and should be prioritized for use (I, A) (17). However, for HFpEF patients, RAAS inhibitors, β receptor blockers and MRAs are still recommended for Class IIa patients. Research has shown that symptoms in HF patients usually appear before cardiac remodeling. For patients with acute myocardial infarction, if LVEF is not significantly reduced, diagnosing new onset HF may face certain difficulties. But for patients with HF episode complicating post-AMI, it is of great significance to explore whether they can benefit from the early application of GDMT for preventing ventricular remodelin (17). For patients who undergo revascularization after AMI, the EMMY study and the PARADISE-MI study suggest that early use of SGLT 2 inhibitors and angiotensin receptor II blocker-neprilysin inhibitor (ARNI) should be considered to improve ventricular remodeling (18, 19). However, further confirmation from RCTs is still needed.

Therefore, patients with HF episode complicating post-AMI in this study were divided into a GDMT group and a no-GDMT group based on whether GDMT was used before discharge to explore the prognostic effect of in-hospital initiation of GDMT on patients with HF episode complicating post-AMI. The 1-year follow-up results showed that the use of GDMT was associated with a lower incidence of all-cause mortality and all-cause readmission composite events, cardiac mortality and cardiac readmission composite events, and all-cause readmission and cardiac readmission. The initiation of GDMT to prevent ventricular remodeling in hospitals can significantly improve the prognosis of patients with HF episode complicating post-AMI. This study grouped patients based on whether GDMT was used before discharge, without an explanation of the changes in GDMT within one year after discharge in either group, which is also a limitation of this real-world cohort study. On the other hand, the use of GDMT during follow-up is influenced by the patient's blood pressure, heart rate, and eGFR, which are dynamic processes. Therefore, this study mainly emphasized the timing of GDMT initiation in the hospital rather than the long-term impact of GDMT on patients with HF episode complicating post-AMI.

The conclusions of this study cannot be extended to all patients with HF episode. The main reason is that HF has more complex and diverse causes (20). The pathophysiology of HF is caused by different etiologies; therefore, different treatment methods are needed. This suggests that research on HF should target patients with different etiologies. In 2020, relevant research proposed the latest classification method for HFpEF, which divides HFpEF into 5 types based on etiology: vascular-related HFpEF, cardiomyopathy-related HFpEF, right heart- and pulmonary-related HFpEF, valvular- and rhythm-related HFpEF, and extracardiac disease-related HFpEF. Vascular disease-related HFpEF can be further subdivided into hypertension-, coronary artery disease-, and coronary microcirculation dysfunction-related HFpEF (7). In addition, previous studies have focused on patients after myocardial infarction. The SAVE-STEMI trial included 200 STEMI patients undergoing PCI and evaluated the efficacy of sacubitril valsartan compared to that of ramipril to explore whether the ARNI can still benefit patients after MI. The results showed that the composite endpoints of cardiac death, MI, and HF hospitalization were significantly reduced in the sacubitril valsartan group, mainly due to a decrease in HF hospitalization at 6 months. At 6 months, patients in the sacubitril valsartan group showed significant improvements in ventricular remodeling indicators such as LVEF, left ventricular end diastolic diameter, and left ventricular end systolic diameter (21). However, the study population did not include HF episode patients after AMI.

The follow-up results of this study showed that the incidence of all-cause death and all-cause readmission composite events, cardiac death and cardiac readmission composite events, all-cause readmission events, and cardiac readmission events within one year after PCI were 11.00%, 7.78%, 10.44%, and 7.40%, respectively. Compared to the incidence of 5.93% (355/5,988) of all cause readmissions within 1 year in the EMPEROR Preserved study, the incidence of all cause readmissions in this study was greater, possibly because the patients included in this study had HF after AMI. Compared with patients with heart failure of other causes, it is difficult for myocardial infarction patients to recover the myocardial contractile function of the infarcted area after reconstruction (16). In addition, studies have shown that the proportion of primary PCI in patients with AMI is approximately 80% (22), but the proportion of primary PCI in this study was only 60%. This may also lead to sustained myocardial ischemia in patients who do not undergo primary PCI, causing an increase in myocardial infarction and thus increasing the incidence of clinical events one year after PCI. In this single center cohort study, although some indicators in the baseline data showed significant differences, Cox regression analysis was used to correct for them. The application of GDMT was associated with a lower incidence of all-cause mortality and all-cause readmission composite events, all-cause readmission events, cardiac mortality and cardiac readmission composite events, and cardiac readmission in patients (*P* < 0.001, *P* < 0.001, *P* = 0.001, *P* < 0.001). A history of atrial fibrillation was associated with a greater incidence of cardiac death and cardiac readmission composite events and cardiac readmission in patients (*P* = 0.012, *P* = 0.011). Related studies have shown that the proportions of females and older individuals among HFpEF patients are greater, and these patients are more likely to have concomitant atrial fibrillation, chronic kidney disease, and noncardiovascular diseases (20). In the Framingham cohort, a history of atrial fibrillation was more strongly correlated with HFpEF than with HFrEF, and a history of HF was associated with a 2-fold increase in the incidence of atrial fibrillation events (23). A subgroup analysis of heart failure patients participating in the EAST study (24) showed that early drug rhythm control strategies have advantages in reducing the risk of cardiovascular events compared to heart rate control (24). Compared with previous trials comparing heart rate control and rhythm control in HFrEF patients, the HF subgroup in this trial was composed mainly of HFpEF and HFmrEF patients (24). In addition to the GDMT and history of atrial fibrillation shown in this study, multiple factors can predict HF episode. Due to the inclusion of a portion of HFpEF in HF episodes, the current scoring system for HFpEF can assist in prediction (25, 26). The H_2_FPEF score is derived and validated using the gold standard reference of invasive hemodynamic measurements. The six components of the H_2_FPEF score include easily accessible information: overweight status, hypertension status, atrial fibrillation status, pulmonary hypertension status, advanced age, and filling pressure (25). A score of 6 or higher indicates the presence of HFpEF. However, this score does not include levels of natriuretic peptide, and therefore, the H_2_FPEF score should be combined with the HFA-PEFF scoring system in clinical practice (26). Notably, in clinical practice, when using BNP/NT proBNp for the diagnosis of heart failure, adjustments should be made to patient age, BMI, eGFR, etc.

Given the various influencing factors mentioned above, this study conducted subgroup analysis based on sex, age, BMI, location of myocardial infarction, and myocardial infarction subtype. The results showed that, except for the lower wall series myocardial infarction subgroup and NSTEMI subgroup, there was no statistically significant difference in the incidence of cardiac death or cardiac readmission composite events or in the incidence of cardiac readmission events between patients in the GDMT group and those in the non-GDMT group (*P* > 0.05). The analysis results for all other subgroups were consistent with the overall results. Previous studies have shown that compared to males, females have a greater LVEF (27), and the overall longitudinal strain preservation of the left ventricle is better (28). Therefore, the likelihood of a decrease in LVEF is lower, and it is expected that the incidence of HF in females will significantly increase. That finding is somewhat different from the results of this study and may also be due to the inclusion of AMI patients in this study, with a greater proportion of male patients with AMI. In previous studies, patients included not only AMI patients but also a history of pregnancy and preeclampsia in females, which may also be associated with an increased risk of hospitalization (29). An increase in the severity of obesity is associated with an increased risk of hospitalization for HF. Despite the obesity paradox observed to some extent in both HFpEF and HFrEF patients, patients with an elevated BMI have an improved survival rate (30, 31). In HFpEF patients, there is a U-shaped relationship between BMI and all-cause mortality, with the lowest incidence of events occurring between BMIs of 32 and 34 kg/m^2^. Although the emergence of this paradox may be due to weight loss in individuals with end-stage HF and obesity-related HF patients developing heart failure at a younger age, these populations still have seemingly better outcomes than older and weaker individuals with a similar severity of HF (32, 33). In addition, a lack of physical activity and obesity are closely related to poor health status and prognosis in patients (34, 35). Weight loss has beneficial effects on heart failure events and exercise tolerance (36). Therefore, although there is a U-shaped relationship between BMI and all-cause mortality, obesity should still be controlled in HF patients.

The subgroup analysis results in this study suggest that there are relatively small differences among different subgroups, and the results are relatively stable. In this study, differences in performance between the lower wall series myocardial infarction subgroup and the NSTEMI subgroup compared to all patients were observed. First, GDMT may yield more pronounced benefits in patients with with heart failure episode complicating post-anterior wall M. Second, the reduced sample size in the subgroup analyses may have limited statistical power to detect significant associations. Previous studies have explored the effects of GDMT based on gender, age, and BMI (37). Further exploration should be conducted on the role of GDMT in improving the prognosis of patients with different infarct sizes, infarct sites, and subtypes of myocardial infarction. Although the infarct size of the two groups of patients was not analyzed in this study, the myocardial infarction size of AMI patients was considered to be related to the early implementation of PCI. Therefore, the baseline data of this study included the door-to-balloon time and the first medical contact-to-balloon time. The results showed no significant difference between the two groups. In the future, objective indicators such as myocardial magnetic resonance imaging are still needed to measure the infarct area to explore their impact on the effectiveness of GDMT in patients with HF after AMI.

This study conducted subgroup analysis based on the use of RAAS inhibitors, β receptor blockers, MRAs, and SGLT2i and revealed that early initiation of β receptor blockers in the hospital and combined use of GDMT have certain significance in improving the clinical prognosis of patients with HF after AMI. For SGLT2i, it may be mainly due to the low proportion of patients receiving SGLT2is in this study. The nonsignificant differences in the use of RAAS inhibitors and MRAs in patients suggest that future research should further explore the impact of RAAS inhibitors or MRAs alone on the prognosis of patients with HF after AMI. On the other hand, compared to the use of one drug in GDMT alone, the combined use of GDMT has a better prognosis for patients. Notably, various guidelines and consensuses provide corresponding guidance on the administration sequence and principles of GDMT in clinical practice. For example, a low-dose drug combination is preferred, and it is generally recommended to increase the dose to the target dose or maximum tolerable dose within 4 weeks. Step-by-step initiation: Using the minimum dose, if some patients still cannot tolerate the simultaneous initiation of the “new quadruple” drug, they can start with 1–2 drugs first. If patients can tolerate this dose, then it should be gradually increased. According to the individualization principle, clinical decisions should be made according to the patient's individual conditions (combined with diabetes, myocardial infarction, renal insufficiency, hyperkalemia, arrhythmia, etc.) and drug characteristics (1, 38). In addition, combining conventional treatment with cardiac rehabilitation is currently a hot research topic, and its benefits for patients can be further explored (39).

## Limitations

The limitations of this study are as follows: first, it is a retrospective study with a small sample size and short follow-up time, resulting in a lower incidence of primary endpoint events, which affects the overall power of this study. The outcome measures in this study were limited to adverse events such as all-cause mortality, all-cause readmission, cardiac death, and cardiac readmission, without analysis of echocardiographic or cardiac magnetic resonance-related parameters beyond one year post-PCI. Although we have comprehensively identified potential confounding factors and adjusted for them using Cox regression analysis, we acknowledge that unmeasured confounders may still exist, which is a common limitation inherent in observational studies. The single-center design has limited the generalizability. Results may not apply to other hospital settings with different patient ethnics, demographics, or treatment protocols. Second, the application of GDMT in patients in this study was subjectively determined by doctors, and the grouping was mainly based on the application of GDMT to patients at discharge. Patients were not randomly grouped; some patients changed their medication regimen during follow-up, and the outcome indicators did not include ventricular remodeling-related indicators. In addition, this study included only patients with HF episode after AMI and did not include patients with HF episode caused by other factors. Future multi-center studies are needed to validate these findings across broader populations.

## Conclusion

For patients with HF episode after AMI who are undergoing PCI, GDMT to prevent ventricular remodeling can reduce the incidence of all-cause mortality and all-cause readmission composite events, cardiac mortality and cardiac readmission composite events, all-cause readmission events and cardiac readmission events and improve the disease prognosis. Notably, these benefits may be more pronounced in the subgroups of patients with inferior wall myocardial infarction, and those with NSTEMI. For such patients, if there are no contraindications, GDMT should be initiated as early as possible before discharge.

## Data Availability

The original contributions presented in the study are included in the article/Supplementary Material, further inquiries can be directed to the corresponding author.
