# Supplementary material for: The effect of guideline-directed medicine on patients with new-onset heart failure following acute myocardial infarction
Source: Front Cardiovasc Med. 2025 Sep 19;12:1639213. doi: 10.3389/fcvm.2025.1639213 (PMC12491194; doi:10.3389/fcvm.2025.1639213)
Supplement: Supplementary file 1 [file Table1.doc]

**Table S1 Medication List**

|  | Starting dose | Target dose |
| --- | --- | --- |
| SGLT2 inhibitor |  |  |
| Dapagliflozin | 10mg o.d. | 10mg o.d. |
| ARNI |  |  |
| Sacubitril/valsartan | 49/51mg b.i.d. | 97/103mg b.i.d |
| ACE-I |  |  |
| Captopril | 6.25mg t.i.d. | 50mg t.i.d. |
| Enalapril | 2.5mg b.i.d. | 10-20mg b.i.d |
| Ramipril | 2.5mg b.i.d. | 5mg b.i.d. |
| ARB |  |  |
| Candesartan | 4mg o.d. | 32mg o.d. |
| Losartan | 50mg o.d. | 150mg o.d. |
| Valsartan | 40mg b.i.d. | 160mg b.i.d. |
| Beta-blockers |  |  |
| Bisoprolol | 1.25mg o.d. | 10mg o.d. |
| Carvedilol | 3.125mg b.i.d. | 25mg b.i.d. |
| Metoprolol succinate (CR/SR) | 12.5-25mg o.d. | 200mg o.d. |
| MRA |  |  |
| Spironolactone | 25mg o.d. | 50mg o.d. |
| CCB |  | |
| Nifedipine | 10-30mg t.i.d. | |
| Nifedipine (SR) | 10-80mg b.i.d. | |
| Nifedipine (CR) | 30-60mg o.d. | |
| Amlodipine | 2.5-10mg o.d. | |
| Levamlodipine | 2.5-5mg o.d. | |
| Felodipine | 2.5-10mg b.i.d. | |
| Felodipine (ER) | 2.5-10mg o.d. | |
| Nitrendipine | 20-60mg t.i.d. | |
| Benidipine | 4-8mg o.d. | |
| Verapamil | 80-480mg b.i.d. | |
| Diltiazem | 90-360mg b.i.d. | |
| Thiazide diuretics |  | |
| Hydrochlorothiazide | 6.25-25mg o.d. | |
| Indapamide | 0.625-2.5mg o.d. | |
| Loop diuretics |  | |
| Furosemide | 20-80mg | |
| Torsemide | 5-10mg | |
| Antiplatelet |  | |
| Aspirin | 75-100mg o.d. | |
| Clopidogrel | 75mg o.d. | |
| Ticagrelor | 90mg b.i.d. | |
| Statins |  | |
| Atorvastatin | 10-80mg o.d. | |
| Rosuvastatin | 5-20mg o.d. | |
| Simvastatin | 20-40mg o.d. | |
| Pitavastatin | 1-4mg o.d. | |

SGLT2=sodium-glucoseco-transporter2; o.d.=omne indie (once daily); ARNI=angiotensin receptor neprilysininhibitor; b.i.d.=bis indie(twice daily); ACE-I=angiotensin-converting enzyme inhibitor; t.i.d.=ter indie (three times a day); ARB=angiotensin receptor blocker; CR=controlled release; SR=sustained release; MRA=mineralocorticoidreceptorantagonist; CCB=calcium channel blocker; ER=extended release.
